# Gambogic Acid Induces Pyroptosis of Colorectal Cancer Cells through the GSDME-Dependent Pathway and Elicits an Antitumor Immune Response

**DOI:** 10.3390/cancers14225505

**Published:** 2022-11-09

**Authors:** Hanjie Xu, Danya Zhang, Rui Wei, Ying Zhou, Geyang Dai, Jie Li, Yue Sun, Fei Li, Ling Xi

**Affiliations:** 1 Cancer Biology Research Center (Key Laboratory of the Ministry of Education), Tongji Hospital, Tongji Medical College, Huazhong University of Science and Technology, Wuhan 430030, China; 2 Department of Gynecologic Oncology, Tongji Hospital, Tongji Medical College, Huazhong University of Science and Technology, Wuhan 430030, China

**Keywords:** colorectal cancer, gambogic acid, pyroptosis, caspase-3/GSDME

## Abstract

**Simple Summary:**

The treatment of colorectal cancer (CRC) is still a major challenge, and the manipulation of pyroptosis has become a novel therapeutic strategy for cancers. Gambogic acid (GA), a common traditional Chinese medicine, has been reported to be a potential agent for cancer treatment. However, the anticancer effects of GA in CRC and the molecular mechanism remain unclear. We conducted this study to solve this problem. The results demonstrated that GA inhibits the viability of CRC cells through GSDME-dependent pyroptosis by regulating the activation of caspase-3. Encouragingly, the process of GA-induced pyroptosis elicits an anti-tumor immune response. These results reveal for the first time that GSDME-dependent pyroptosis is a previously unrecognized mechanism by which GA inhibits CRC, and these findings have important implications for GA use in the treatment of cancers.

**Abstract:**

Pyroptosis is a recently identified form of programmed cell death (PCD) that exerts a vital influence on the antitumor immune response. GA, a xanthone structure isolated from gamboge resin, is a naturally occurring bioactive ingredient with several anticancer activities, such as activities that affect cell cycle arrest, apoptosis, and autophagy. Here, we found that GA decreased the viability of the CRC cell lines, HCT116 and CT26, in a dose- and time-dependent manner, and multiple pores and large bubbles in the membranes, which are morphological characteristics of pyroptosis, were observed by light microscopy and transmission electron microscopy (TEM). Furthermore, the cleavage of gasdermin E (GSDME) was observed after exposure to GA, along with concomitant activation of caspase-3. Additionally, GSDME-dependent pyroptosis triggered by GA could be attenuated by siRNA-mediated knockdown of GSDME and treatment with the caspase-3 inhibitor. Moreover, we found that GA induced pyroptosis and significantly inhibited tumor growth in CT26 tumor-bearing mice. Strikingly, significantly increased proportions of CD3^+^ T cells, cytotoxic T lymphocytes (CTLs), and dendritic cells (DCs) were observed in the tumor microenvironment in the GA-treated groups. Moreover, significantly increased proportions of CTLs and effector memory T cells (TEM) (CD8^+^ CD44^+^ CD62L^−^) were also detected in the spleens of the GA-treated groups, suggesting that the pyroptosis-induced immune response generated a robust memory response that mediated protective immunity. In this study, we revealed a previously unrecognized mechanism by which GA induces GSDME-dependent pyroptosis and enhances the anticancer immune response. Based on this mechanism, GA is a promising antitumor drug for CRC treatment that induces caspase-3-GSDME-dependent pyroptosis. This study provides novel insight into cancer chemoimmunotherapy.

## 1. Introduction

CRC is the third most common and third most lethal malignancy worldwide, with an estimated 1.9 million new cases and 0.93 million related deaths in 2020 [1]. Due to the lack of obvious symptoms, most CRC patients are diagnosed at an advanced stage, and it is extremely difficult to cure CRC with surgery at this stage [2]. Thus, chemotherapy has become an integral part of the comprehensive therapeutic regimens used to treat advanced CRC [3]. However, CRC chemotherapy treatment is still not ideal due to an unsatisfactory response rate and serious side effects [4,5]. Therefore, new and more effective approaches for the treatment of CRC are urgently needed to enhance the benefits of chemotherapy. Increasing evidence indicates that the regulation of pyroptosis holds great promise and therapeutic potential for a variety of malignant tumors [6,7,8].

Pyroptosis, a new form of PCD distinguished from apoptosis, is characterized by the formation of large bubbles in the plasma membrane [9]. Pyroptosis can be activated through the canonical caspase-1 inflammasome pathway or the noncanonical caspase-4/5/11 pathway [10,11,12]. Activated caspases cleave gasdermin D (GSDMD) to release its N-terminal domain, which induces pyroptosis by forming transmembrane pores in cellular membranes [13]. Some recent studies demonstrated that GSDME, another member of the gasdermin family, can mediate a switch from chemotherapy-induced caspase-3-mediated apoptosis to pyroptosis [6,14]. The GSDME N-terminal fragment (GSDME-N), which is produced via cleavage by activated caspase-3, also forms transmembrane pores to cause pyroptosis, which is characterized by the formation of large bubbles and the release of lactate dehydrogenase (LDH) [6]. This finding brings a completely new concept that activated caspase-3 can trigger pyroptosis by cleaving GSDME. Strikingly, recent studies have shown that pyroptosis is associated with the modulation of cancer immunity, which provides important insight for cancer chemoimmunotherapy [15].

**Figure 1:**
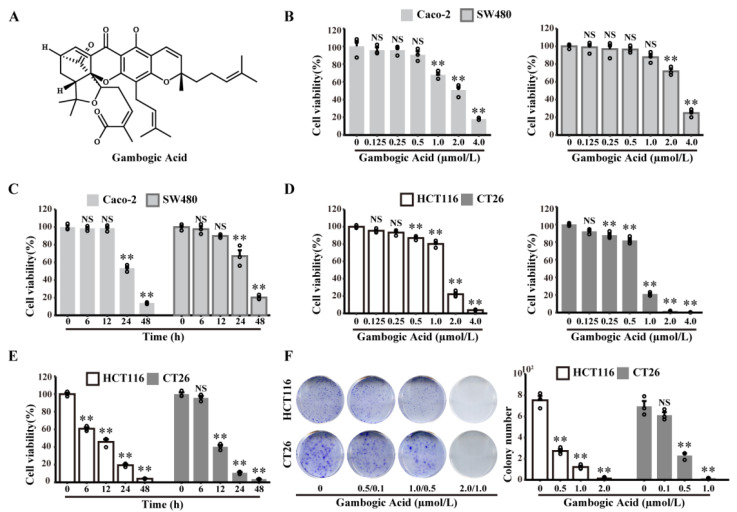
GA decreases Caco-2, SW480, HCT116, and CT26 cell viability in dose- and time-dependent manners. (**A**) Chemical structure of GA. (**B**) Caco-2, SW480, (**D**) HCT116, and CT26 cells were treated with GA (0–4 μmol/L) for 24 h, and cell viability was analysed by the CCK8 assay. (**C**) Caco-2 and SW480 cells were treated with GA (2 μmol/L) for different times, and cell viability was analysed by the CCK8 assay. (**E**) HCT116 and CT26 cells were treated with GA (2 μmol/L and 1 μmol/L, respectively) for different times, and cell viability was analysed by the CCK8 assay. (**F**) HCT116 and CT26 cells were treated with the indicated concentrations of GA and stained with crystal violet. The cells were exposed to 0.1% DMSO as control. All experiments were repeated three times, and results were expressed as mean ± SD. NS, not significant, ** *p* < 0.01 vs. control.

Gambogic acid (GA, chemical formula: C_38_H_44_O_8_) is the major active ingredient isolated from Gamboge, which is a brownish resin that is secreted from Garcinia hanburyi, as shown in Figure 1A [16]. GA exerts multiple anticancer effects in cancers, such as the induction of apoptosis, autophagy, cell cycle arrest, and proliferation [17]. GA triggers mitochondria-dependent apoptosis in JeKo-1 cells through the modulation of Bcl-2 and Bax [18]. In MCF-7 cells, GA can exert the anticancer effects by downregulating the MDM2 oncogene and upregulating the expression of p21Waf1/CIP1 with or without p53 activation [19]. Moreover, GA has known antiproliferative and proapoptotic activities in CRC cells [20,21]. Notably, GA exhibits more cytotoxicity towards cancer cells than normal cells [22,23]. These studies indicated that GA is a promising natural compound for cancer therapy. However, the anticancer effects of GA on CRC in vivo and the molecular mechanism underlying its anticancer effects remain unclear.

Here, we aimed to explore the effects of GA in CRC, and to further elucidate the underlying molecular mechanism; in particular, we focused on the ability of GA to induce cancer cell pyroptosis and regulate the antitumor immune response to combat CRC.

## 2. Materials and Methods

### 2.1. Cell Cultures

The human CRC cell line Caco-2, SW480, HCT116, and mouse CT26 cells were purchased from ATCC, and were cultured in L-15 (PYG0038, Boster, Wuhan, China), Eagle’s minimum essential medium (30-2003, ATCC, Manassas, VA, USA), McCoy’s 5A (M4892, Sigma-Aldrich, St. Louis, MO, USA) and RPMI 1640 medium (Gibco BRL, Carlsbad, CA, USA) supplemented with 10% or 20% FBS (every green). The cells were incubated in 5% CO_2_. After incubation for 24 h, the cells were pretreated with or without the caspase-3 inhibitor Z-DEVD-FMK (20 μM, 210344-95-9, MedChemExpress, Monmouth Junction, USA) for 3 h, and then different doses of GA (S2448, Selleck chemicals, Shanghai, China) were added and incubated for another 12 h. Since GA was dissolved in DMSO and stored at −20 °C, cells exposed to DMSO (0.1%) were used as a control in all of the in vitro experiments.

### 2.2. Cell Viability Assays

Cell viability was monitored with cell counting kit-8 (CCK-8) assay (Dojindo Laboratories, Kyushu Island, Japan). In brief, HCT116 and CT26 cells were separately seeded into 96-well plates at 2500 cells/well and treated with different concentrations of GA for different time intervals. Then, cell viability was determined by CCK-8 assay according to the manufacturer’s protocol.

### 2.3. ATP and LDH Release Assay

The intracellular ATP level was measured based on assessment of the bioluminescent luciferin-luciferase reaction with an ATP Assay Kit (S0027, Beyotime). After different treatments, the medium in the well was aspirated, and the cells were lysed. Subsequently, the cell lysates were centrifuged, and the supernatants were collected for further experiments. One hundred microlitres of ATP detection reagent was added and incubated for 5 min. Then, 20 μL of sample was added and mixed quickly. The luminescence of the samples was determined by a Synergy 2 multifunction microplate reader and used to calculate the ATP levels.

The LDH release assay was performed using an LDH assay kit (C0017, Beyotime). The cell supernatants were collected after exposure to various treatments or LDH release reagents, which were used to generate samples with maximum enzyme activity. Subsequently, 120 μL of supernatant was added to 96-well plates and LDH detection reagents (60 μL) and incubated for 30 min in the dark after mixing. The absorbance (OD490) was measured by a microplate reader (SpectraMax ABS Plus).

### 2.4. Annexin V-FITC/PI Assay

To evaluate cell death mechanisms, cell apoptosis assays were conducted. HCT116 and CT26 cells were treated with GA for 12 h with or without Z-DEVD-FMK. Subsequently, the cells were digested and collected for Annexin V-FITC/PI (556547, BD Bioscience,) staining, and then monitored using a Beckman Coulter flow cytometer.

### 2.5. RNA Sequencing (RNA-Seq)

RNA sequencing was performed on HCT116 and CT26 cells. The HCT116 and CT26 cells were treated with either 2 μmol/L or 1 μmol/L GA for 12 h, and the control cells were exposed to 0.1% DMSO. Total RNA was extracted with TRIzol reagent, and libraries were constructed. Subsequently, the libraries were sequenced on an Illumina Novaseq 6000 platform to generate 150 bp paired-end reads. The clean reads were mapped to the reference genome using HISAT2, and fragments per kilobase million (FPKM) of each gene were calculated. Then, the read counts of each gene were obtained by HTSeq-count.

### 2.6. Western Blotting Analysis

The treated cells or tumor tissues were harvested for total protein extraction. Samples with equal amounts of protein were separated by 10% SDS-PAGE under denaturing conditions and then transferred to polyvinylidene difluoride (PVDF) membranes. Subsequently, the membranes were blocked with 5% bovine serum albumin (BSA) (GC305006, Servicebio, Wuhan, China), and then incubated with primary antibodies at 4 °C overnight. The primary antibodies were as follows: anti-human GSDMD (1:1000, ab215203, Abcam, Boston, MA, USA), anti-mouse GSDMD (1:1000, ab209845, Abcam, Boston, MA, USA), anti-GSDME (1:1000, ab215191, Abcam, Boston, MA, USA), anti-caspase-3 (1:1000, #9662, Cell Signaling Technology, Danvers, MA, USA), anti-Bax (1:2000, ab32503, Abcam, Boston, MA, USA), anti-Bcl-2 (1:2000, ab182858, Abcam, Boston, MA, USA). An anti-rabbit secondary antibody (1:20000, ANT020, Antejie Biotechnology, Wuhan, China) was incubated for 1 h at room temperature after the membranes were washed. Subsequently, immunoreactive bands were detected using an enhanced chemiluminescence (ECL) kit (Advansta) and observed by a multimode chemiluminescence system (Bio-Rad, Hercules, CA, USA). Then, after washing with stripping solution, the membranes were incubated with anti-GAPDH (1:10000, A19056, ABclonal, Wuhan, China) to normalize the expression levels of proteins in the same sample. Densitometry analysis was processed with ImageJ software (version 1.52V, Bethesda, Rockville, MD, USA).

### 2.7. SiRNA-Mediated Knockdown

HCT116 and CT26 cells were seeded into 6-well plates (1 × 10^5^/well) for siRNA-mediated knockdown. After 24 h in culture, 2 μL of GSDME (RiboBio, Guangzhou, China) or control siRNA (RiboBio) was transfected with Lipo3000 according to the manufacturer’s instructions. After 72 h, transfected HCT116 and CT26 cells were treated with GA or Z-DEVD-FMK for subsequent experiments.

### 2.8. Immunohistochemistry

The tumor tissues, hearts, livers, spleens, lungs, and kidneys of mice were harvested, fixed in 4% paraformaldehyde, and embedded in paraffin. Serial 4 μm-thick sections were obtained for haematoxylin and eosin (H&E) or immunohistochemistry. For immunohistochemistry, after deparaffinization and antigen retrieval, 3% H_2_O_2_ was used to remove endogenous peroxidase, and 5% BSA blocking buffer was used to block nonspecific antibody binding. Subsequently, the slides were incubated with primary antibodies overnight at 4 °C. The primary antibodies were as follows: anti-Ki67 (1:500, GB111499, Servicebio, Wuhan, China), anti-CD3 (1:150, ab16669, Abcam, Boston, MA, USA), and anti-CD8 (1:2000, ab217344, Abcam, Boston, MA, USA). An immunohistochemical staining kit (SP9000, ZSGB Bio, Beijing, China) was used to label primary antibodies, and then, the slides were visualized by diaminobenzidine (DAB), and counterstained with haematoxylin.

### 2.9. In Vivo Anticancer Therapy

Five- to six-week-old BALB/c mice were purchased from Gempharmatech Co., Ltd (Nanjing, China) and were maintained in the Animal Experimental Center of Tongji Hospital, Tongji Medical College, Huazhong University of Science and Technology (HUST). CT26 cells (approximately 2 × 10^5^) were subcutaneously injected into the right flanks of BALB/c mice. On day 7 after post-tumor implantation, the mice were randomly divided into three groups (six in each group): a control group, a low-dose group, and a high-dose group. In the low-dose group and high-dose group, 2 and 8 mg/kg GA were intravenously administered via the tail vein every two days, and the control group mice were treated with vehicle (5% DMSO + 95% L-arginine solution (0.53 g/L); i.v.). The body weights and tumor volumes were recorded every two days. On the 11th day, the mice were sacrificed 48 h after the last administration, and blood samples were collected. Then, the tumor and major organs were harvested, weighed, and analysed via H&E, immunohistochemistry, western blotting, and flow cytometry. The tumor volume was calculated based on the following formula: Tumor volume = length × width^2^ × 0.5.

### 2.10. Antitumor Immune Response Analysis

Fresh tumors and spleen tissues were collected to analyse immunocytes via FACS. Briefly, tumor tissues were dissociated using Mouse Tumor Dissociation Kits (130-096-730, Miltenyi, Bergisch-Gladbach, GER). Next, the cell suspensions were filtered through a 70 μm nylon cell strainer. A 40% Percoll solution was used to resuspend the cell pellets, and an 80% Percoll solution was layered over them. Then, the samples were centrifuged for 35 min at 524 g. After that, the cells were resuspended and washed with PBS. Then, all the cells were stained with Fixable Viability Stain 700 (564997, BD Biosciences, Franklin Lakes, NJ, USA) for live/dead identification. Subsequently, anti-CD16/32 antibodies were added to block the nonspecific Fc receptor, and relevant antibodies were added and incubated at 4 °C for 30 min. The following antibodies were purchased from BD Biosciences and used in the study: anti-CD8a-FITC (553030), anti-CD11c-PerCP-Cy5.5 (560584), anti-CD44-PE-Cy7 (560569), anti-CD62L-APC (553152), anti-CD45-APC-Cy7 (557659), anti-CD3e-BV421 (562600), anti-I-A/I-E-V500 (562366), and anti-CD4-BV605 (563151). The cells were resuspended in PBS to remove the excess antibodies and resuspended in cell staining buffer. The cells were filtered using a 200-mesh cell strainer and analysed by flow cytometry. Additionally, immunohistochemistry staining for CD3 and CD8 was performed to further evaluate antitumor immune effects.

### 2.11. Statistical Analysis

All experiments were conducted at least three times in vitro. Statistical analyses were performed in the R software environment (version 4.1.2, R Core Team, R Foundation for Statistical Computing, Vienna, Austria), using the “ggpubr” R package (https://mirror.lzu.edu.cn/CRAN/bin/windows/contrib/4.1/ggpubr_0.4.0.zip, accessed on 15 August 2022) and the “DescTools” R package (https://mirror.lzu.edu.cn/CRAN/bin/windows/contrib/4.1/DescTools_0.99.47.zip, accessd on 21 October 2022). The differences among groups were evaluated using one-way analysis of variance (ANOVA), and comparisons between two groups were evaluated by Bonferroni post hoc test. A *p*-value less than 0.05 was considered statistically significant.

## 3. Results

### 3.1. The Effects of GA on CRC Cells Viability

To evaluate the anticancer effects of GA on CRC cells, a CCK8 assay was performed with human or mouse CRC cell lines (Caco-2, SW480, HCT116, or CT26, respectively). As described in Figure 1B,D, GA exerted potent anticancer activity against CRC cells in a dose-dependent manner. Based on these results, we treated SW480, Caco-2, and HCT116 cells with 2 μmol/L GA, and CT26 cells with 1 μmol/L GA in the subsequent time-course studies. The results indicated that GA exerted anticancer activity in a time-dependent manner (Figure 1C,E). To further confirm the anticancer activity of GA, a colony formation assay was performed, and the results further confirmed the potent dose-dependent anticancer effect of GA (Figure 1F). In summary, these data clearly demonstrated that GA could decrease the viability of either HCT116 or CT26 cells in a dose- and time-dependent manner.

### 3.2. The Abilities of GA to Induce Pyroptosis in CRC Cells

**Figure 2:**
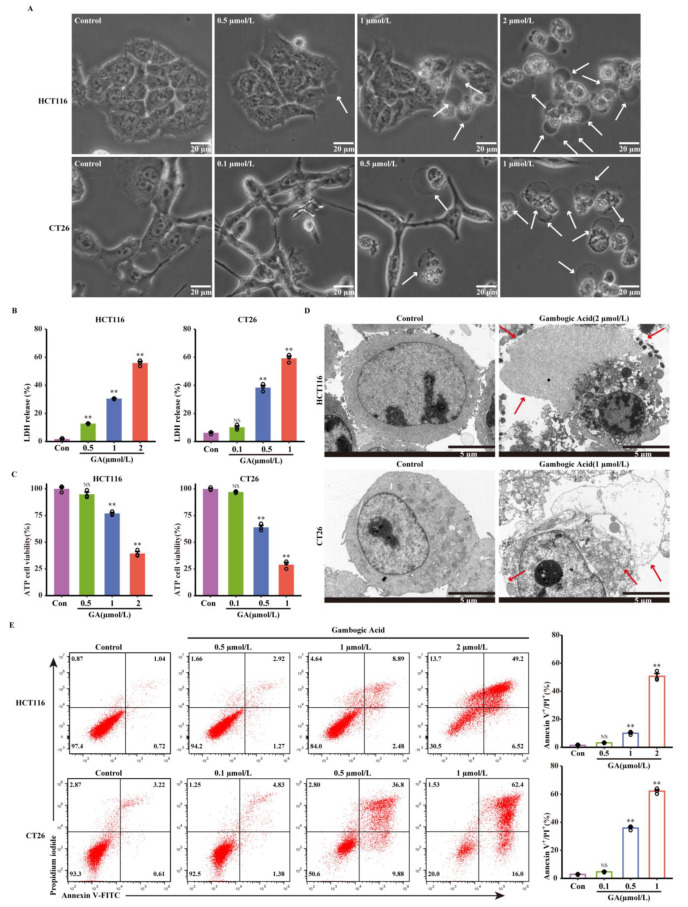
GA triggered cell pyroptosis in CRC cells. (**A**) Representative bright-field images of HCT116 and CT26 cells treated with GA for 12 h. The white arrows point to pyroptotic cells. Scale bar: 20 μm. (**B**) The release of LDH and (**C**) ATP cell viability after treatment with GA for 12 h. (**D**) Representative transmission electron microscopy images of HCT116 and CT26 cells treated with GA (2 μmol/L or 1μmol/L) for 12 h. The red arrows indicate the large bubbles, cell membrane breaks, and organelle edema. Scale bar: 5 μm. (**E**) Flow cytometric analysis of HCT116 and CT26 cells treated with GA for 12 h. The cells were exposed to 0.1% DMSO as control. All experiments were repeated three times and results were expressed as mean ± SD. Con, control. NS, not significant, ** *p* < 0.01 vs. control.

Since decreased cell viability can also be caused by pyroptosis, the ability of GA to trigger pyroptosis was investigated in this study. First, morphological alterations were monitored in HCT116 and CT26 cells after exposure to GA at different concentrations. As demonstrated in Figure 2A, the obvious morphological alterations, including large bubbles from the cellular membrane and cellular swelling, were observed in response to GA treatment; these changes were distinct from classic morphological changes observed during apoptosis and were consistent with changes in cell morphology during pyroptosis. Classic pyroptotic morphological features, including large bubble structure formation and cellular membrane rupture, were also observed by TEM, as shown in Figure 2D. Second, to verify cell membrane rupture and intracellular content leakage, adenosine triphosphate (ATP) cell viability assays and LDH release assays were performed. As shown in Figure 2B,C, HCT116 and CT26 cells treated with GA released LDH and exhibited decreased intracellular ATP levels. In pyroptotic cells, propidium iodide (PI) can enter cells through pores that form in the cellular membrane and then stain the nuclei [24]. Thus, the pyroptosis of these cells was further investigated by flow cytometry analyses of Annexin V-FITC and PI staining. The flow cytometry analysis results showed that the number of Annexin V-FITC/PI positive HCT116 and CT26 cells was increased in response to GA exposure in a dose-dependent manner (Figure 2E). Collectively, these results indicated that GA could induce the pyroptosis of HCT116 and CT26 CRC cells.

### 3.3. RNA Sequencing and Analysis

**Figure 3:**
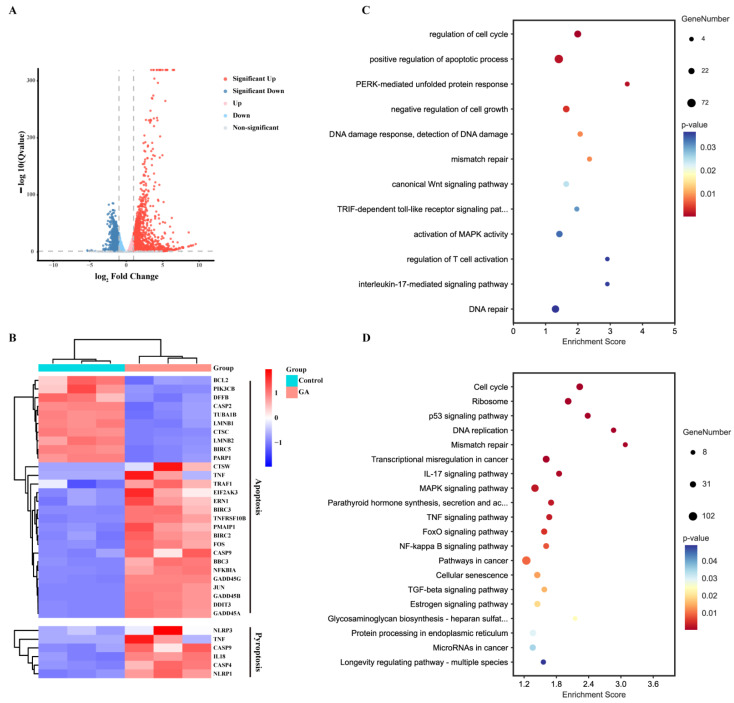
RNA sequencing analysis. (**A**) Volcano plots of differentially expressed genes of HCT116 cells in response to 2 μmol/L GA for 12 h. (**B**) Heatmap analysis of differentially expressed genes involved in apoptosis and pyroptosis. (**C**) GO and (**D**) KEGG enrichment in GA-treated HCT116 cells and control cells. The cells were exposed to 0.1% DMSO as control.

To further explore the abilities of GA to induce pyroptosis and related immune responses, RNA sequencing of HCT116 and CT26 cells was performed. Compared with the control group (|log2 fold change| > 1 and adjusted *p*-value < 0.05), 2943 genes were differentially expressed in the GA-treated HCT116 cells, including 1816 upregulated genes and 1127 downregulated genes, and 904 upregulated genes and 507 downregulated genes were detected in the GA-treated CT26 cells (Figure 3A and Figure S1A). Then, the association of differentially expressed genes with programmed cell death (including apoptosis and pyroptosis) was analysed, and obvious gene changes were observed and confirmed that GA was able to activate apoptosis and pyroptosis pathways in CRC (Figure 3B and Figure S1B). Furthermore, Gene Ontology (GO) and Kyoto Encyclopedia of Genes and Genomes (KEGG) pathway enrichment analyses indicated that after GA treatment, the differentially expressed genes were enriched in apoptosis and inflammatory signaling pathways, such as the apoptosis signaling pathway, p53 signaling pathway, FoxO signaling pathway, MAPK signaling pathway, TGF-beta signaling pathway, inflammatory response, and TNF signaling pathway (Figure 3C,D and Figure S1C,D).

### 3.4. GSDME but Not GSDMD Is Involved in GA-Triggered Pyroptosis Medicated by the Activation of Caspase-3

**Figure 4:**
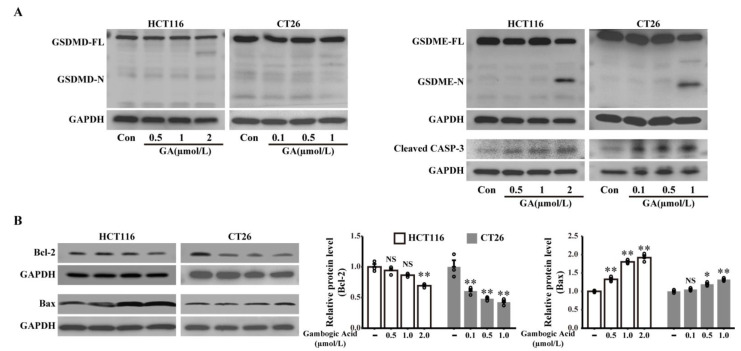
GSDME, rather than GSDMD, was involved in GA-induced pyroptosis in CRC cells. (**A**) Expressions of GSDMD, GSDME, and cleaved caspase-3 in HCT116 and CT26 cells treated with GA for 12 h were analysed by western blotting. (**B**) The expressions of Bcl-2 and Bax in HCT116 and CT26 cells treated with GA were analyzed by western blotting. The cells were exposed to 0.1% DMSO as control. All experiments were repeated three times and results were expressed as mean ± SD. CASP-3, caspase-3, Con, control, NS: not significant. NS *p* > 0.05 vs. control, * *p* < 0.05 vs. control, ** *p* < 0.01 vs. control. The whole western blots were shown in Figure S3.

Both GSDMD and GSDME, which are important pyroptosis substrates, can be cleaved by activated caspases to trigger pyroptosis [6,9,25]. Thus, to investigate whether GSDMD and GSDME are required for GA- triggered pyroptosis, we examined the expression of these proteins and their N-terminal domains in HCT116 or CT26 cells after treatment with different doses of GA. As demonstrated in Figure 4A, western blotting analysis showed that GSDMD was expressed in HCT116 and CT26 cells, but no cleavage of GSDMD was observed. The results indicated that GSDMD is not involved in GA-induced pyroptosis in HCT116 or CT26 cells.

GSDME, a newly identified pyroptotic executive protein, was found to be cleaved by activation of caspase-3 and triggered pyroptosis in response to various chemotherapy agents [6]. Thus, GSDME and GSDME-N expression was also assessed by western blotting analysis, and the results indicated that GA treatment caused GSDME-N release, which is a marker of pyroptosis, in CRC cells (Figure 4A). Furthermore, a dose-dependent induction of cleaved caspase-3 (activated) was also observed after GA treatment (Figure 4A). Moreover, the expression levels of apoptosis-related proteins (Bcl-2 and Bax) were detected by western blotting. After GA exposure, a decreased Bcl-2 expression along with increased Bax expression was found in HCT116 and CT26 cells, indicating that GA-induced activation of caspase-3 might be related to Bcl-2 and Bax (Figure 4B). Collectively, these data suggest that caspase-3-mediated GSDME cleavage might participate in GA-induced pyroptosis in CRC cells.

### 3.5. Knocking down GSDME Inhibited GA-Induced Pyroptosis

**Figure 5:**
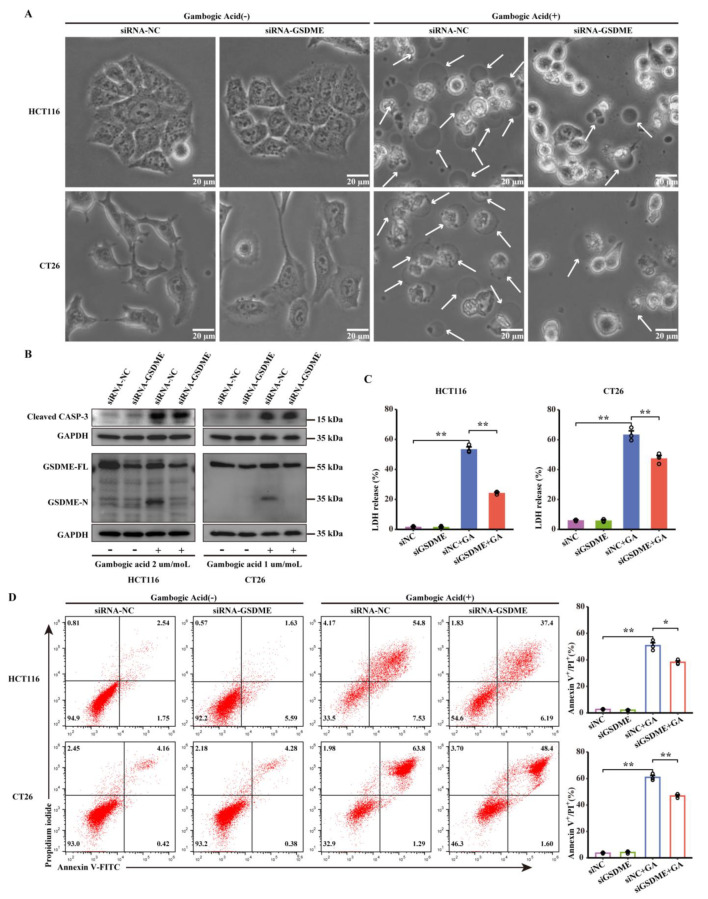
GSDME mediated pyroptosis of CRC cells in response to GA treatment. HCT116 and CT26 cells were transfected with siGSDME or siNC and then treated with GA (2 μmol/L or 1 μmol/L) for 12 h. (**A**) SiNC- or siGSDME-infected HCT116 and CT26 cells were treated with GA, and representative bright-field images were obtained. The white arrows point to pyroptotic cells. (**B**) Expression of GSDME and cleaved caspase-3 in siNC- or siGSDME-transfected HCT116 and CT26 cells treated with GA analysed by western blotting. The whole western blots were shown in Figure S4. (**C**) The release of LDH and (**D**) flow cytometric analysis were performed after treatment with GA. The cells were exposed to 0.1% DMSO as control. All experiments were repeated three times and results were expressed as mean ± SD. NC, negative control. * *p* < 0.05 vs. control, ** *p* < 0.01 vs. control.

The abovementioned results, namely, the cleavage of GSDME, demonstrated that GSDME might be indispensable for GA-induced pyroptosis in CRC cells. To confirm our speculation, knockdown of GSDME expression was conducted by siRNA against GSDME. The effects of siRNA targeting GSDME were confirmed by western blotting analysis, and the expression of GSDME was weakly attenuated in HCT116 and CT26 cells. Nonetheless, GSDME-N was still barely detected in GSDME-knockdown cells after GA treatment (Figure 5B). However, GSDME-specific siRNA had no effect on GA-mediated caspase-3 activation compared with that of negative control (NC) siRNA, confirming that GSDME was a downstream signaling molecule of activated caspase-3 in GA-induced pyroptosis. Interestingly, inconsistent with the typical pyroptotic morphology of NC siRNA-transfected cells treated with GA, GSDME-knockdown cells showed shrinkage and non-lytic cell death without the formation of large bubbles in the cellular membrane, indicating that GSDME-knockdown inhibits GA-induced pyroptosis (Figure 5A). Consistent with the morphological changes observed in GSDME-knockdown cells, GSDME-knockdown markedly inhibited the GA-induced release of LDH (Figure 5C). Furthermore, the flow cytometry results demonstrated that GSDME deficiency resulted in fewer annexin V-FITC/PI double positive HCT116 and CT26 cells after treatment with GA (Figure 5D). In summary, these results confirmed that GSDME is a crucial executioner of GA-triggered pyroptosis in CRC cells.

### 3.6. GA-Induced Pyroptosis was Dependent on Activation of Caspase-3

**Figure 6:**
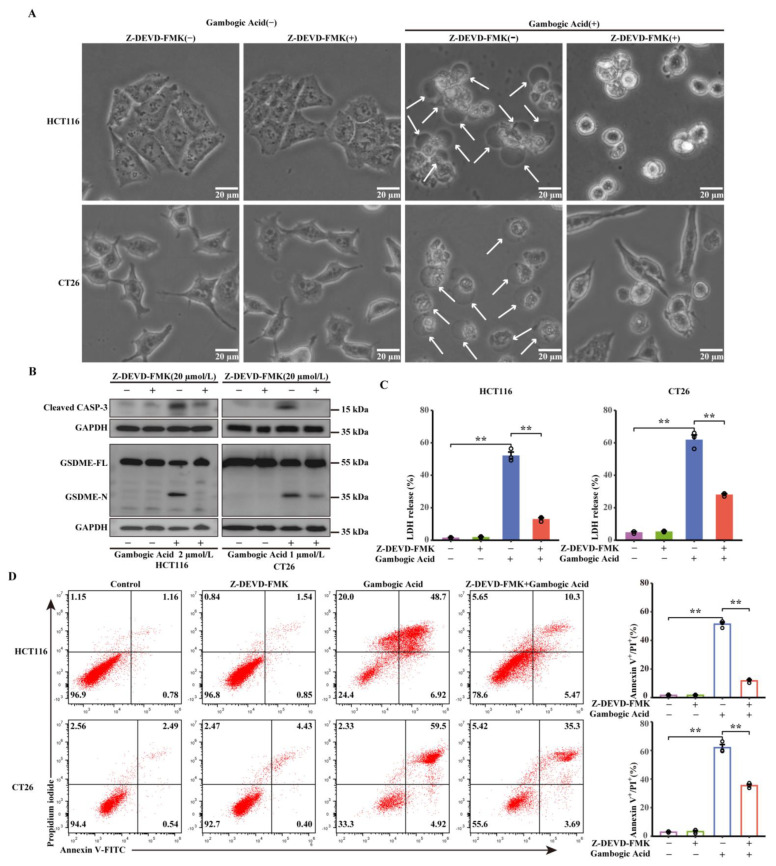
Caspase-3 is essential for GSDME-dependent pyroptosis. (**A**) Representative bright-field images of HCT116 and CT26 cells after treatment with GA (2 μmol/L or 1 μmol/L, 12 h) in the presence or absence of Z-DEVD-FMK (20 μmol/L). The white arrows point to pyroptotic cells. (**B**) GSDME and Cleaved caspase-3 expression levels were analysed by western blotting. The whole western blots were shown in Figure S5. (**C**) The release of LDH and (**D**) flow cytometric analysis were performed after treatment of GA in the presence or absence of Z-DEVD-FMK (20 μmol/L). The cells were exposed to 0.1% DMSO as control. All experiments were repeated three times and results were expressed as mean ± SD. ** *p* < 0.01 vs. control.

Activation of caspase-3 was observed in CRC cells treated with GA (Figure 4A), suggesting that activated caspase-3 may be responsible for the cleavage of GSDME. To investigate this, Z-DEVD-FMK, a caspase-3 inhibitor, was used to inhibit caspase-3 activity in HCT116 and CT26 cells before GA treatment. First, changes in cell morphology were observed, and the results demonstrated that the GA-induced morphological features of pyroptosis were abrogated by Z-DEVD-FMK (Figure 6A). Furthermore, western blotting was performed to determine whether caspase-3 is responsible for the cleavage of GSDME. We found that Z-DEVD-FMK treatment led to a visible decrease in GSDME-N generation (Figure 6B), further confirming that activated caspase-3 was essential for the cleavage of GSDME. Additionally, significant decreases in LDH release and the number of annexin V-FITC/PI double positive cells were also detected in HCT116 and CT26 cells pretreated with Z-DEVD-FMK (Figure 6C,D). Taken together, these results suggest that GSDME-dependent pyroptosis was triggered by GA in CRC cells, which was dependent on the activation of caspase-3.

### 3.7. GA Inhibited Tumor Growth and Induced CRC Cell Pyroptosis In Vivo

**Figure 7:**
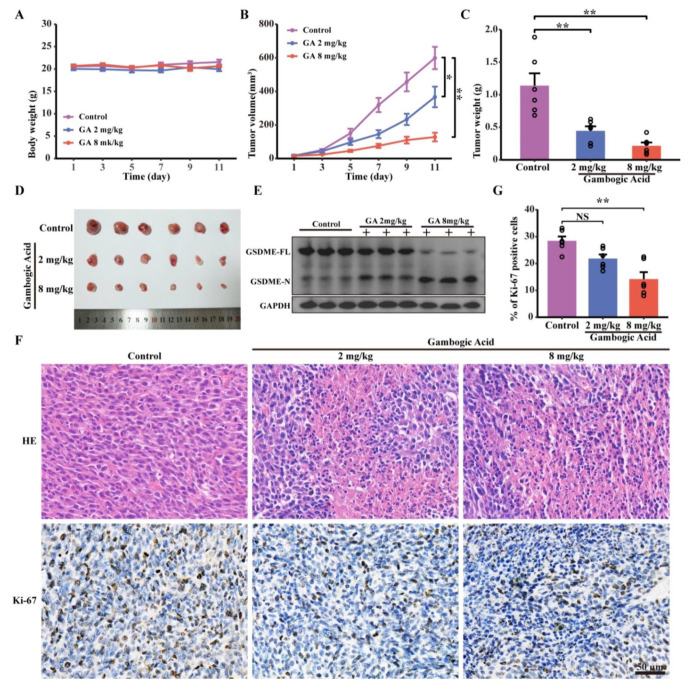
GA inhibited the tumor growth and triggered pyroptosis of CRC cells in vivo. CT26 cells were inoculated into BALB/c mice to establish the murine xenograft model. (**A**) Change curves of mice weight after GA treatments (*n* = 6). (**B**) Tumor volume, (**C**) tumor weight, (**D**) photographs of isolated tumor after GA treatment. (**E**) Western blotting analyses of GSDME-FL and GSDME-N expression in treated tumor tissues. The whole western blots were shown in Figure S6. (**F**) H&E staining and Ki-67 immunohistochemistry analysis of tumor tissues after GA treatments. (**G**) Quantitation of Ki-67 staining from immunohistochemical analysis. NS, not significant, * *p* < 0.05 vs. control, ** *p* < 0.01 vs. control.

To further verify the anticancer activity of GA in vivo, murine CT26 tumor-bearing mouse models were established. As shown in Figure 7B–D, the treatment with GA at both the low-dose and high-dose significantly reduced the volumes and weights of CRC solid tumors. The average of tumor weight decreased from 1.139 g (control group) to 0.445 g (low-dose group), or 0.214 g (high-dose group) (Figure 7C). Furthermore, IHC staining for Ki-67, which is a marker of proliferation, demonstrated that GA inhibited the proliferation of CRC tumors in vivo, as indicated by the decreased expression of Ki-67 (Figure 7F,G). The body weights were also measured, and the results indicated that GA treatment had no effect on the body weights of mice compared with those in the control group (Figure 7A). Additionally, to further determine whether GA-induced pyroptosis is involved in the reduction in tumor volume, the expression of GSDME was evaluated. Western blot analyses revealed that GA upregulated the expression of GSDME-N, suggesting that GA-mediated GSDME-dependent pyroptosis participates in GA-triggered cell death in vivo (Figure 7E).

In addition, the levels of serological markers of liver and kidney injury, including alanine aminotransferase (ALT), aspartate aminotransferase (AST), urea, and creatinine (Cr), were measured to assess the toxicity and response to GA treatment. The results indicated that compared with the control group, the levels of ALT and AST were significantly higher in the GA groups but were within the normal range, and no differences in the urea and Cr levels were observed among all the groups (Figure S2A). Furthermore, the major organs (heart, liver, spleen, lung, and kidney) from the three groups were harvested and subjected to H&E staining. As shown in Figure S2B, no obvious signs of toxicity were detected in the major organs of the GA treatment groups and the control group. These results indicated that GA is a safe chemotherapeutic agent for the treatment of CRC and that it does not damage normal organs in mice.

### 3.8. Antitumor Effect of GA Via the Pyroptosis-Induced Immune Response

**Figure 8:**
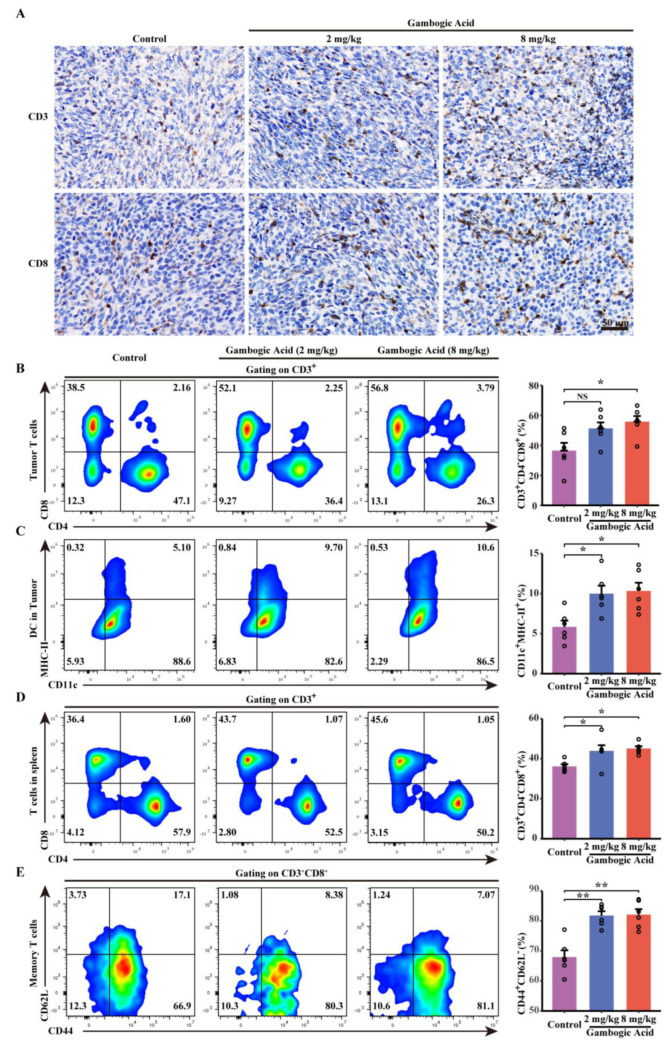
GA-induced pyroptosis regulates the tumor immune microenvironment. (**A**) CD3 and CD8 immunohistochemistry analysis of tumor tissues after GA treatments. (**B**) Representative flow cytometric analysis and quantitative analysis of proportions of CD8^+^ and CD4^+^ T cells gating on CD3^+^ cells within the tumor tissues. (**C**) Representative flow cytometric analysis that GA treatment significantly increased the proportions of CD3^+^ and CD8^+^ T cells in tumor tissues (**A**) and quantitative analysis the proportion of CD11c+MHC-II+ cells within the tumor tissues. (**D**) Representative flow cytometric analysis and quantitative analysis of the proportions of CD8^+^ and CD4^+^ T cells gating on CD3^+^ cells in the spleen. (**E**) Representative flow cytometric analysis and quantitative analysis the proportion of effector memory T cells (TEM) (CD44^+^ CD62L^−^) gating on CD3^+^ CD8^+^ cells in the spleen. NS *p* > 0.05 vs. control, * *p* < 0.05 vs. control, ** *p* < 0.01 vs. control.

Based on pore-forming activity, tumor cells undergoing pyroptosis release cellular contents, including proinflammatory molecules and tumor-associated antigens, into the tumor microenvironment. These processes are conducive to regulating the tumor immune microenvironment and activating the immune response; therefore, pyroptosis induction has been considered an efficient strategy to promote chemotherapy-activated antitumor immunity. To determine the effect of GA on immune response, immunohistochemistry was performed, and the results visually demonstrated that GA treatment significantly increased the number of of CD3^+^ and CD8^+^ T cells in tumor tissues (Figure 8A).

Consistent with the immunohistochemistry results, further flow cytometric analysis demonstrated an increased proportion of CTLs (CD3^+^ CD8^+^) within the tumor microenvironment in both the low-dose and high-dose GA groups (Figure 8B). Furthermore, to investigate changes in the immune response after GA treatment, we further assessed dendritic cells (DCs) in the tumor microenvironment, and the results indicated that GA treatment could enhance CD11c^+^MHC-II^+^ dendritic cell infiltration (Figure 8C). In addition, we also monitored the proportion of CTLs in the spleen and found that the CTLs proportion was higher in the GA therapy groups than in the control group (Figure 8D). Interestingly, we also observed that GA could lead to a significantly increased proportion of effector memory T cells (TEM) (CD8^+^ CD44^+^ CD62L^−^) in the spleen, and these cells can be efficient for creating a robust memory response that mediates protective immunity (Figure 8E). These results indicated that GA could initiate T-cell clone expansion, generate memory T cells, and trigger the antitumor immune response.

## 4. Discussion

This study demonstrated that GA, which is a candidate anticancer agent that is isolated from Gamboge, significantly inhibited CRC by triggering pyroptosis. We further revealed a previously unrecognized mechanism underlying the anticancer effects of GA on CRC: the induction of caspase-3/GSDME-dependent pyroptosis and the regulation of the antitumor immune response.

Apoptosis is generally considered to be the main form of PCD that is responsible for the efficacy of tumor treatments [26,27]. Numerous studies have indicated that GA exerts potent anticancer effects by inducing apoptosis in various tumor types [23,28,29]. The induction of apoptosis by GA has also been confirmed in CRC cells [30]. In the present study, we extended the conventional views on the molecular mechanisms underlying the anticancer activity of GA and proposed that GSDME-dependent pyroptosis contributed to the effects of GA on CRC; this conclusion was supported by subsequent evidence. First, GA-treated CRC cells exhibited the formation of balloon-like bubbles, which is a specific morphological feature of pyroptosis. Pyroptosis depends upon the formation of pores in cell membrane by oligomerized proteins. Hence, during pyroptosis, pores open in the cell membrane, leading to the leakage of cellular contents and the release of LDH and ATP; these pores also allow annexin V/PI to enter cells and stain the phospholipid phosphatidylserine (PS) on the inner side of the membrane [31]. Accordingly, LDH release assays, ATP release assays, and Annexin V-FITC/PI staining were conducted to further confirm the occurrence of GA-induced pyroptosis, and the results indicated that GA could induce pyroptosis in HCT116 and CT26 cells. It should be noted that released ATP is unstable at 37 °C. Hence, we measured intracellular ATP levels to indirectly assess ATP release. Additionally, because some other forms of cell death also allow annexin V/PI to enter cells, more sensitive and specific markers to confirm the occurrence of pyroptosis are still urgently needed.

GSDMD-N, which is a pyroptosis executive protein that is generated by the cleavage of GSDMD by activated caspase-1/-4/-5/-11, forms transmembrane pores and triggers pyroptosis [25]. In addition to GSDMD, another gasdermin protein family member (GSDME) can be cleaved by activated caspase-3, and N-terminal-cleaved GSDME can also lead to pyroptosis of cancer cells [6]. In our study, the expression of both GSDMD and GSDME was observed in CRC cells, but only the cleavage of GSDME was observed after GA treatment. The signaling interrelation between apoptosis and pyroptosis indicated that these two forms of PCD occur simultaneously and reciprocally regulate each other to inhibit cancer cells after treatment with chemotherapy drugs [14,32]. The induction of apoptosis by GA has also been confirmed in CRC cells [20,21]. Consistently, our results showed that activated caspase-3 was also observed in GA-treated CRC cells, suggesting that a conceivable interaction between pyroptosis and apoptosis might occur in GA-treated CRC cells. RNA sequencing analysis showed that expression of genes related to pyroptosis and apoptosis was significantly changed, and the differentially expressed genes were also enriched in apoptosis-related signaling pathways. Furthermore, GSDME knockdown did not fully reverse cell death and had no effect on the GA-mediated activation of caspase-3; these results also demonstrated that apoptosis might participate in GA-induced cell death. Subsequently, we found that GA treatment caused a decrease in the anti-apoptotic protein Bcl-2 and an increase in the pro-apoptotic protein Bax in both HCT116 and CT26 cells. Considering these results, we hypothesized that the changes of apoptosis-related Bcl-2 family proteins activated caspase-3, consequently induced the cleavage of GSDME and ultimately triggered pyroptosis in CRC cells.

To validate the above hypothesis, siRNA technology was performed to knock down the expression of GSDME. GSDME knockdown not only diminished the pyroptotic morphology and LDH release induced by GA but also decreased the proportion of Annexin V-FITC/PI positive cells, indicating that GSDME is crucial for GA-triggered pyroptosis. Additionally, we found that GSDME knockdown did not affect GA-mediated activation of caspase-3, indicating that GSDME is a downstream molecule of activated caspase-3. Although GSDME knockdown significantly decreased the percentage of Annexin V-FITC/PI positive cells, this cell population was not completely diminished, which might be because GSDME was incompletely knocked down and GSDME knockdown resulted in a switch from pyroptosis to apoptosis during GA-induced cell death [14]. Moreover, the caspase-3 inhibitor inhibited the cleavage of GSDME and GA-induced pyroptosis, revealing that the activation of caspase-3 was indispensable for the cleavage of GSDME and GA-induced pyroptosis.

Owing to the strong anticancer effects of GA observed in cellular experiments, BALB/c mice bearing CT26 tumor xenografts were used to further assess the anticancer effect of GA in vivo. GA significantly inhibited tumor growth in CT26 cell xenograft-bearing BALB/c mice, similar to 5-fluorouracil (5-FU) [33]. However, 5-FU, which is an effective and commonly used anticancer agent for the treatment of colon cancer, has side effects that may cause damage to the liver and immunological function [34,35]. In our study, GA exhibited excellent anticancer activity in vivo without causing injury to the major organs or hepatorenal dysfunction; thus, GA might be a safe and effective anticancer chemotherapeutic agent for CRC treatment.

In addition to directly inducing tumor cell death, pyroptotic cells can release inflammatory cytokines and modulate cancer immunity [15]. Furthermore, it has been reported that the pyroptosis of tumor cells triggers anticancer immune responses by promoting DC maturation and increasing T-cell clone expansion [36,37]. Our study demonstrated that GA treatment induced the generation of GSDME-N fragments by cleaving GSDME-FL, which confirmed that pyroptosis was involved in GA-mediated tumor suppression in vivo. Consistent with the findings of others, compared with the control group, the GA-treated groups exhibited higher proportions of CTLs and DCs in the tumor microenvironment [36,37,38]. Moreover, the proportions of CD8^+^ T cells and TEM cells (CD8^+^ CD44^+^ CD62L^−^) in the spleen were also increased in the GA groups. In addition, GO and KEGG enrichment pathways were also related to the inflammatory signaling pathways. Based on these results, GA therapy may regulate the antitumor immune response and trigger adaptive immunological responses. The related mechanism by which the antitumor immune response is modulated is of interest and should be further investigated in our next project.

## 5. Conclusions

Our results demonstrated that GA might be a promising agent for the treatment of CRC. The mechanism underlying these effects was further investigated, and the findings indicated that GA can inhibit CRC cells in vitro and in vivo by triggering caspase-3/GSDME-dependent pyroptosis, which further triggers an antitumor immune response that inhibits tumor growth. These results provide new insight into GSDME-dependent pyroptosis as a previously unrecognized mechanism by which GA inhibits CRC, and these findings have important implications for the development of chemotherapeutic strategies and tumor immunotherapy.

## Data Availability

The data presented in this study is available on request from the corresponding authors.

## Supplementary Materials

The following supporting information can be downloaded at: https://www.mdpi.com/article/10.3390/cancers14225505/s1, Figure S1: RNA sequencing analysis of CT26 cells. (**A**) Volcano plots of differentially expressed genes of CT26 cells in response to 1 μmol/L GA for 12 h. (**B**) Heatmap analysis of differentially expressed genes involved in apoptosis and pyroptosis. (**C**) GO and (**D**) KEGG enrichment in GA-treated CT26 cells and control cells. The cells were exposed to 0.1% DMSO as control. Figure S2: Safety analysis of GA treatment in vivo. (**A**) Biomedical parameters including ALT, AST, UREA and CR at the endpoint of the observation. (**B**) H&E staining analysis of major organs (heart, liver, spleen, lung, kidney) after GA treatments. NS *p* > 0.05 vs. control, * *p* < 0.05 vs. control, ** *p* < 0.01 vs. control. Figure S3: Original western blots of Figure 4. Figure S4: Original western blots of Figure 5B. Figure S5: Original western blots of Figure 6B. Figure S6: Original western blots of Figure 7E.

## References

[1] Sung H., Ferlay J., Siegel R.L., Laversanne M., Soerjomataram I., Jemal A., Bray F. (2021). Global Cancer Statistics 2020: GLOBOCAN Estimates of Incidence and Mortality Worldwide for 36 Cancers in 185 Countries. CA Cancer J. Clin..

[2] Fahy B.N. (2014). Follow-up after curative resection of colorectal cancer. Ann. Surg. Oncol..

[3] Sonbol M.B., Mountjoy L.J., Firwana B., Liu A.J., Almader-Douglas D., Mody K., Hubbard J., Borad M., Ahn D.H., Murad M.H. (2020). The Role of Maintenance Strategies in Metastatic Colorectal Cancer: A Systematic Review and Network Meta-analysis of Randomized Clinical Trials. JAMA Oncol..

[4] Van der Jeught K., Xu H.C., Li Y.J., Lu X.B., Ji G. (2018). Drug resistance and new therapies in colorectal cancer. World J. Gastroenterol..

[5] Wang Q., Shen X., Chen G., Du J. (2022). Drug Resistance in Colorectal Cancer: From Mechanism to Clinic. Cancers.

[6] Wang Y., Gao W., Shi X., Ding J., Liu W., He H., Wang K., Shao F. (2017). Chemotherapy drugs induce pyroptosis through caspase-3 cleavage of a gasdermin. Nature.

[7] Wu D., Wang S., Yu G., Chen X. (2021). Cell Death Mediated by the Pyroptosis Pathway with the Aid of Nanotechnology: Prospects for Cancer Therapy. Angew. Chem. Int. Ed. Engl..

[8] Gaikwad S.M., Phyo Z., Arteaga A.Q., Gorjifard S., Calabrese D.R., Connors D., Huang J., Michalowski A.M., Zhang S., Liu Z.G. (2020). A Small Molecule Stabilizer of the MYC G4-Quadruplex Induces Endoplasmic Reticulum Stress, Senescence and Pyroptosis in Multiple Myeloma. Cancers.

[9] Ding J., Wang K., Liu W., She Y., Sun Q., Shi J., Sun H., Wang D.C., Shao F. (2016). Pore-forming activity and structural autoinhibition of the gasdermin family. Nature.

[10] Lamkanfi M., Dixit V.M. (2014). Mechanisms and functions of inflammasomes. Cell.

[11] Shi J., Zhao Y., Wang Y., Gao W., Ding J., Li P., Hu L., Shao F. (2014). Inflammatory caspases are innate immune receptors for intracellular LPS. Nature.

[12] Hagar J.A., Powell D.A., Aachoui Y., Ernst R.K., Miao E.A. (2013). Cytoplasmic LPS activates caspase-11: Implications in TLR4-independent endotoxic shock. Science.

[13] Sborgi L., Ruhl S., Mulvihill E., Pipercevic J., Heilig R., Stahlberg H., Farady C.J., Muller D.J., Broz P., Hiller S. (2016). GSDMD membrane pore formation constitutes the mechanism of pyroptotic cell death. EMBO J..

[14] Rogers C., Fernandes-Alnemri T., Mayes L., Alnemri D., Cingolani G., Alnemri E.S. (2017). Cleavage of DFNA5 by caspase-3 during apoptosis mediates progression to secondary necrotic/pyroptotic cell death. Nat. Commun..

[15] Hsu S.K., Li C.Y., Lin I.L., Syue W.J., Chen Y.F., Cheng K.C., Teng Y.N., Lin Y.H., Yen C.H., Chiu C.C. (2021). Inflammation-related pyroptosis, a novel programmed cell death pathway, and its crosstalk with immune therapy in cancer treatment. Theranostics.

[16] Liesenklas W., Auterhoff H. (1966). The constitution of gambogic acid and its isomerization. 4. Chemistry of gum-resin. Arch. Pharm. Ber. Dtsch. Pharm. Ges..

[17] Liu Y., Chen Y., Lin L., Li H. (2020). Gambogic Acid as a Candidate for Cancer Therapy: A Review. Int. J. Nanomed..

[18] Xu J., Zhou M., Ouyang J., Wang J., Zhang Q., Xu Y., Xu Y., Zhang Q., Xu X., Zeng H. (2013). Gambogic acid induces mitochondria-dependent apoptosis by modulation of Bcl-2 and Bax in mantle cell lymphoma JeKo-1 cells. Chin. J. Cancer Res..

[19] Rong J.J., Hu R., Qi Q., Gu H.Y., Zhao Q., Wang J., Mu R., You Q.D., Guo Q.L. (2009). Gambogic acid down-regulates MDM2 oncogene and induces p21(Waf1/CIP1) expression independent of p53. Cancer Lett..

[20] Wen C., Huang L., Chen J., Lin M., Li W., Lu B., Rutnam Z.J., Iwamoto A., Wang Z., Yang X. (2015). Gambogic acid inhibits growth, induces apoptosis, and overcomes drug resistance in human colorectal cancer cells. Int. J. Oncol..

[21] Zhang H., Lei Y., Yuan P., Li L., Luo C., Gao R., Tian J., Feng Z., Nice E.C., Sun J. (2014). ROS-mediated autophagy induced by dysregulation of lipid metabolism plays a protective role in colorectal cancer cells treated with gambogic acid. PLoS ONE.

[22] Yang Y., Yang L., You Q.D., Nie F.F., Gu H.Y., Zhao L., Wang X.T., Guo Q.L. (2007). Differential apoptotic induction of gambogic acid, a novel anticancer natural product, on hepatoma cells and normal hepatocytes. Cancer Lett..

[23] Shi X., Chen X., Li X., Lan X., Zhao C., Liu S., Huang H., Liu N., Liao S., Song W. (2014). Gambogic acid induces apoptosis in imatinib-resistant chronic myeloid leukemia cells via inducing proteasome inhibition and caspase-dependent Bcr-Abl downregulation. Clin. Cancer Res..

[24] Miao E.A., Rajan J.V., Aderem A. (2011). Caspase-1-induced pyroptotic cell death. Immunol. Rev..

[25] Shi J., Zhao Y., Wang K., Shi X., Wang Y., Huang H., Zhuang Y., Cai T., Wang F., Shao F. (2015). Cleavage of GSDMD by inflammatory caspases determines pyroptotic cell death. Nature.

[26] McKnight J.J., Gray S.B., O’Kane H.F., Johnston S.R., Williamson K.E. (2005). Apoptosis and chemotherapy for bladder cancer. J. Urol.

[27] Makin G., Dive C. (2001). Apoptosis and cancer chemotherapy. Trends Cell Biol..

[28] Su S.C., Chen Y.T., Hsieh Y.H., Yang W.E., Su C.W., Chiu W.Y., Yang S.F., Lin C.W. (2022). Gambogic Acid Induces HO-1 Expression and Cell Apoptosis through p38 Signaling in Oral Squamous Cell Carcinoma. Am. J. Chin. Med..

[29] Peng W., Chen B.A. (2018). Gambogic acid induces cell apoptosis through endoplasmic reticulum stress triggered inhibition of Akt signaling pathways in extranodal NK/T-cell lymphoma cells. Chin. J. Nat. Med..

[30] Wei J., Yang P., Li W., He F., Zeng S., Zhang T., Zhong J., Huang D., Chen Z., Wang C. (2017). Gambogic acid potentiates the chemosensitivity of colorectal cancer cells to 5-fluorouracil by inhibiting proliferation and inducing apoptosis. Exp. Ther. Med..

[31] Yu P., Zhang X., Liu N., Tang L., Peng C., Chen X. (2021). Pyroptosis: Mechanisms and diseases. Signal. Transduct. Target. Ther..

[32] Yu J., Li S., Qi J., Chen Z., Wu Y., Guo J., Wang K., Sun X., Zheng J. (2019). Cleavage of GSDME by caspase-3 determines lobaplatin-induced pyroptosis in colon cancer cells. Cell Death Dis..

[33] Deng S., Hu B., An H.M., Du Q., Xu L., Shen K.P., Shi X.F., Wei M.M., Wu Y. (2013). Teng-Long-Bu-Zhong-Tang, a Chinese herbal formula, enhances anticancer effects of 5--Fluorouracil in CT26 colon carcinoma. BMC Complement. Altern. Med..

[34] Wu Y., Deng Z., Wang H., Ma W., Zhou C., Zhang S. (2016). Repeated cycles of 5-fluorouracil chemotherapy impaired anti-tumor functions of cytotoxic T cells in a CT26 tumor-bearing mouse model. BMC Immunol..

[35] Wang C., Huo X., Gao L., Sun G., Li C. (2017). Hepatoprotective Effect of Carboxymethyl Pachyman in Fluorouracil-Treated CT26-Bearing Mice. Molecules.

[36] Xiao Y., Zhang T., Ma X., Yang Q.C., Yang L.L., Yang S.C., Liang M., Xu Z., Sun Z.J. (2021). Microenvironment-Responsive Prodrug-Induced Pyroptosis Boosts Cancer Immunotherapy. Adv. Sci..

[37] Liu Y., Lu Y., Ning B., Su X., Yang B., Dong H., Yin B., Pang Z., Shen S. (2022). Intravenous Delivery of Living Listeria monocytogenes Elicits Gasdmermin-Dependent Tumor Pyroptosis and Motivates Anti-Tumor Immune Response. ACS Nano.

[38] Fan J.X., Deng R.H., Wang H., Liu X.H., Wang X.N., Qin R., Jin X., Lei T.R., Zheng D., Zhou P.H. (2019). Epigenetics-Based Tumor Cells Pyroptosis for Enhancing the Immunological Effect of Chemotherapeutic Nanocarriers. Nano Lett..

