# Supplementary material for: Gambogic Acid Induces Pyroptosis of Colorectal Cancer Cells through the GSDME-Dependent Pathway and Elicits an Antitumor Immune Response
Source: Cancers (Basel). 2022 Nov 9;14(22):5505. doi: 10.3390/cancers14225505 (PMC9688471; doi:10.3390/cancers14225505)
Supplement: Supplementary file 1 [file cancers-14-05505-s001.zip › cancers-1949117-supplementary.pdf]

Supplementary Materials

Gambogic Acid Induces Pyroptosis of Colorectal Cancer Cells through the GSDME-Dependent Pathway and Elicits an Antitumor Immune Response

Hanjie Xu [1,2], Danya Zhang [1,2], Rui Wei [1,2], Ying Zhou [1,2], Geyang Dai [1,2], Jie Li [1,2], Yue Sun [1,2], Fei Li [1,2,*] and Ling Xi [1,2,*]

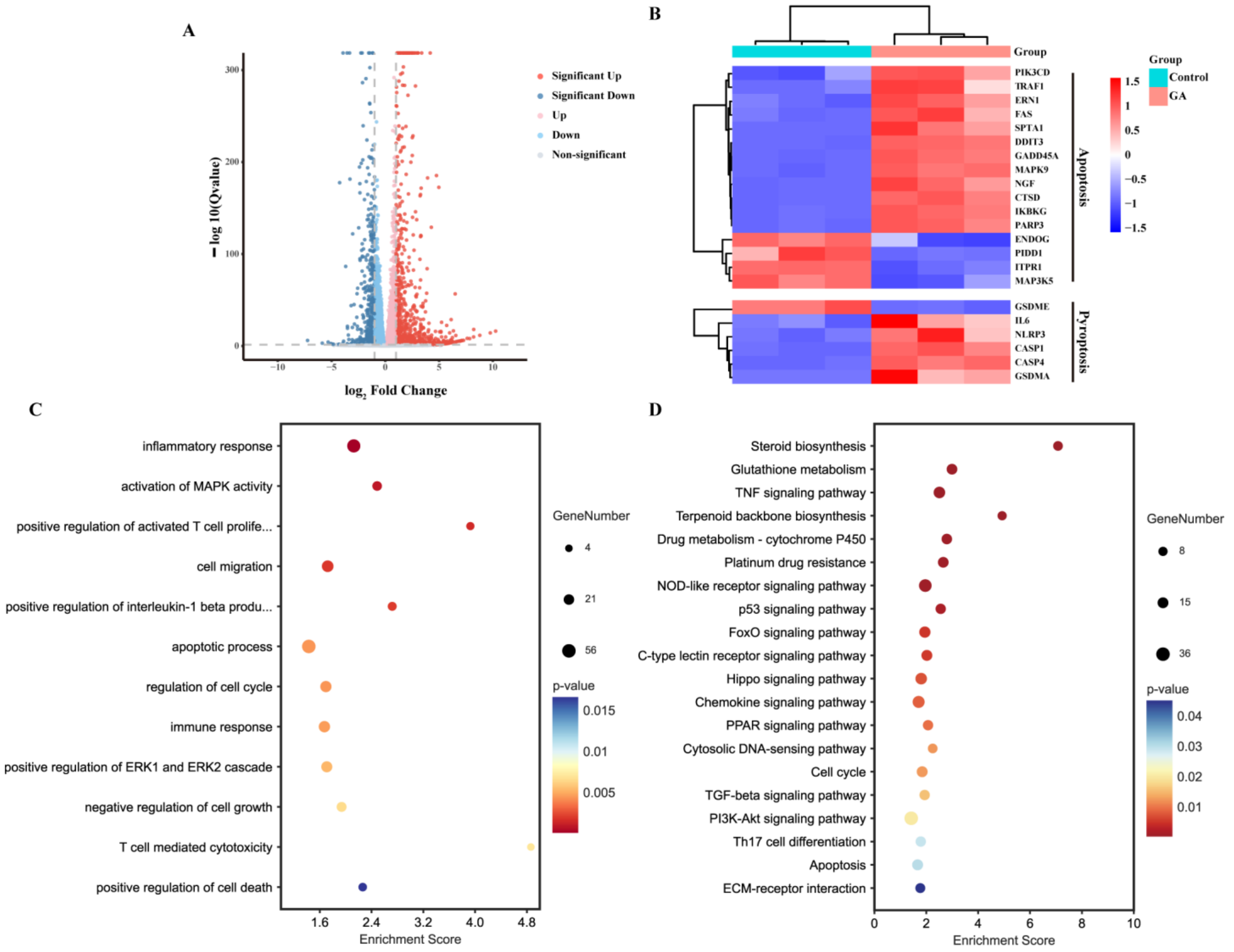

Figure S1. RNA sequencing analysis of CT26 cells. (A) Volcano plots of differentially expressed genes of CT26 cells in response to 1 μmol/L GA for 12 h. (B) Heatmap analysis of differentially expressed genes involved in apoptosis and pyroptosis. (C) GO and (D) KEGG enrichment in GA-treated CT26 cells and control cells. The cells were exposed to 0.1% DMSO as control.

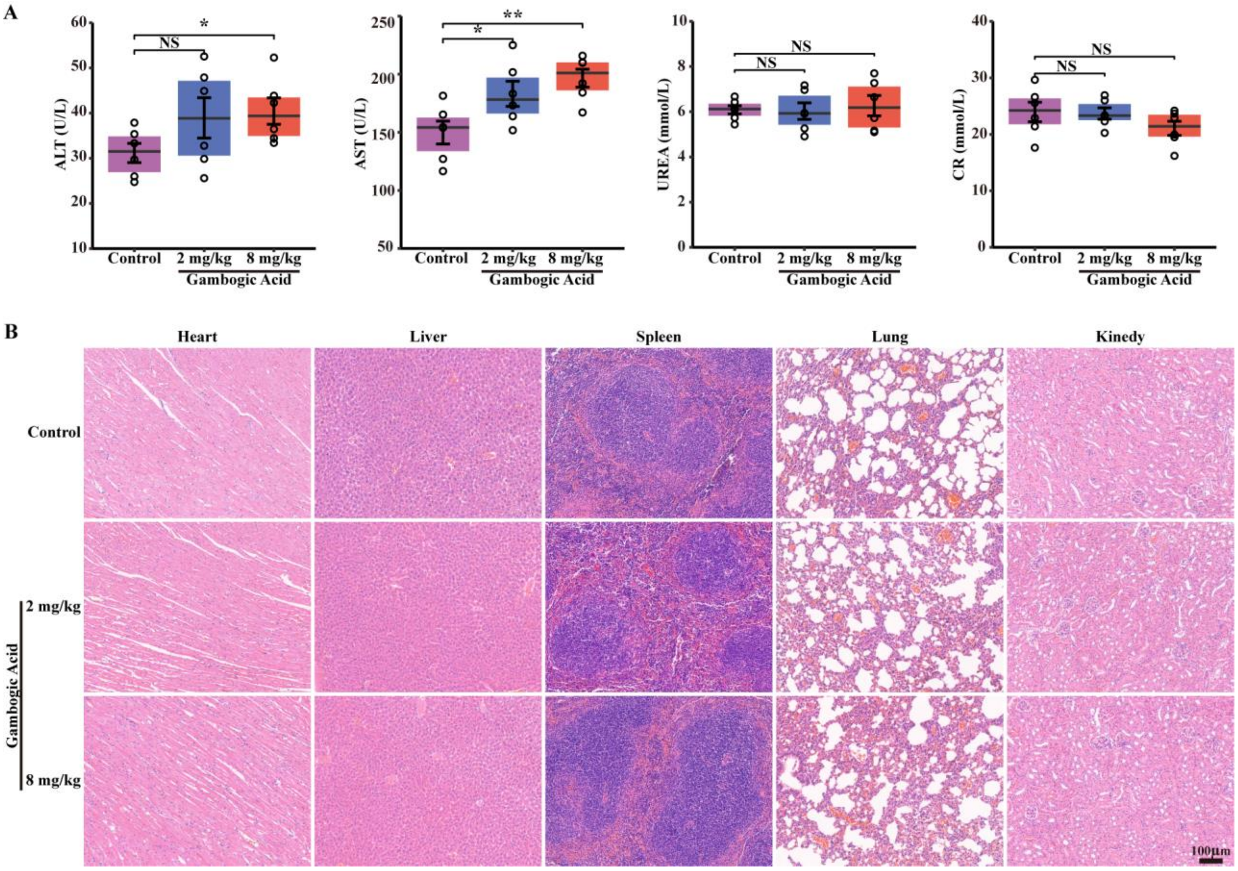

Figure S2. Safety analysis of GA treatment in vivo. (A) Biomedical parameters including ALT, AST, UREA and CR at the endpoint of the observation. (B) H&E staining analysis of major organs (heart, liver, spleen, lung, kidney) after GA treatments. NS p > 0.05 vs. control, * p < 0.05 vs. control, ** p < 0.01 vs. control.

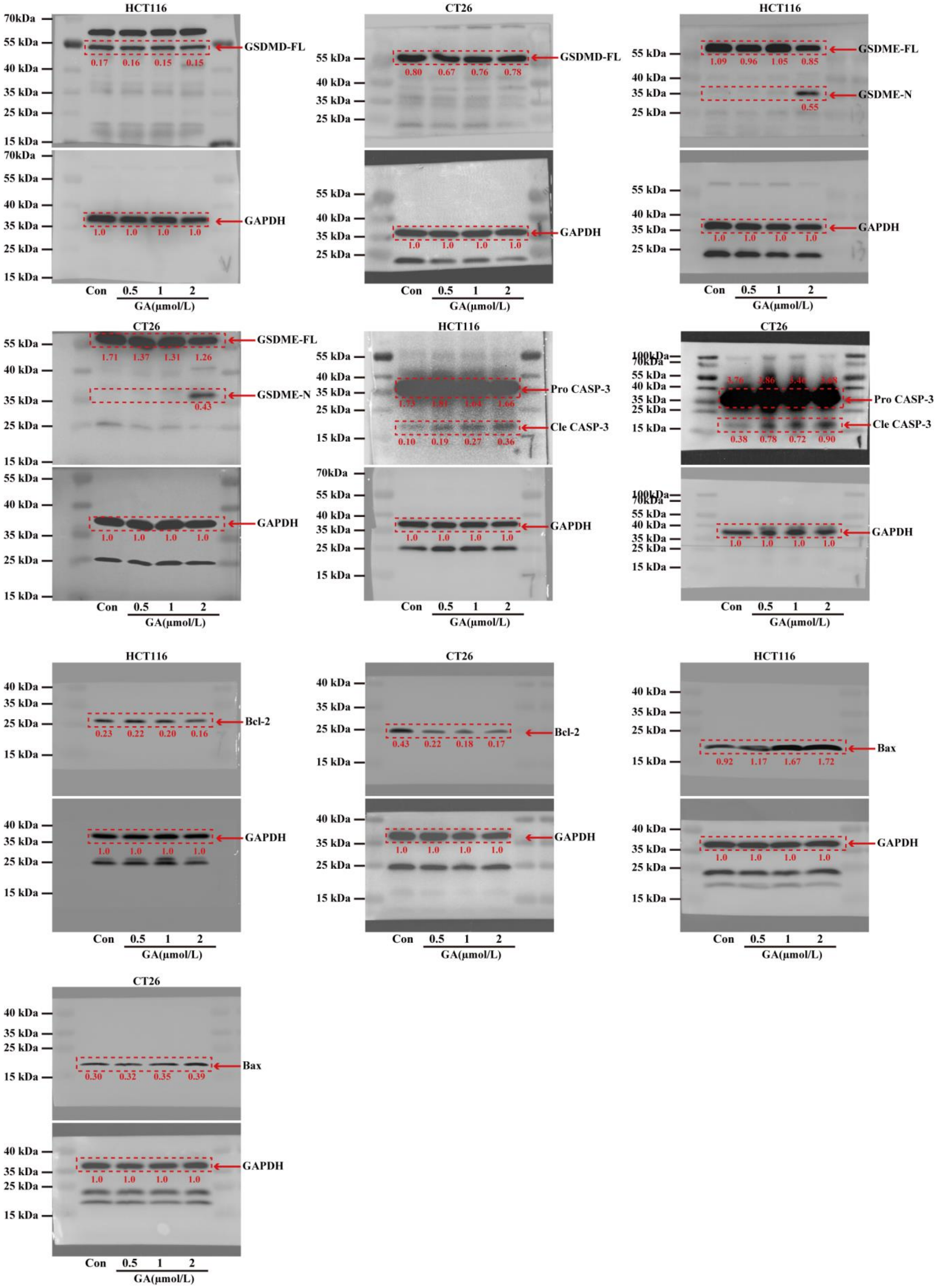

Figure S3. Original western blots of Figure 4.

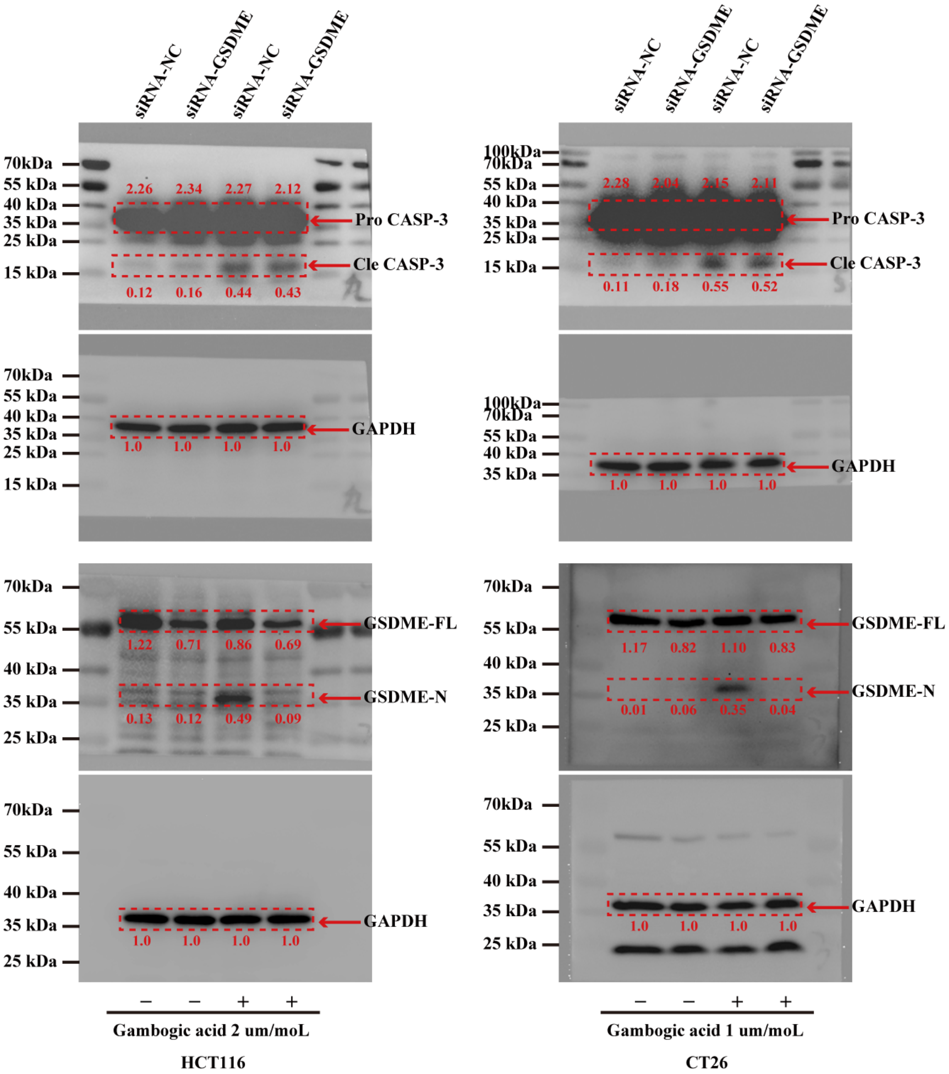

Figure S4. Original western blots of Figure 5B.

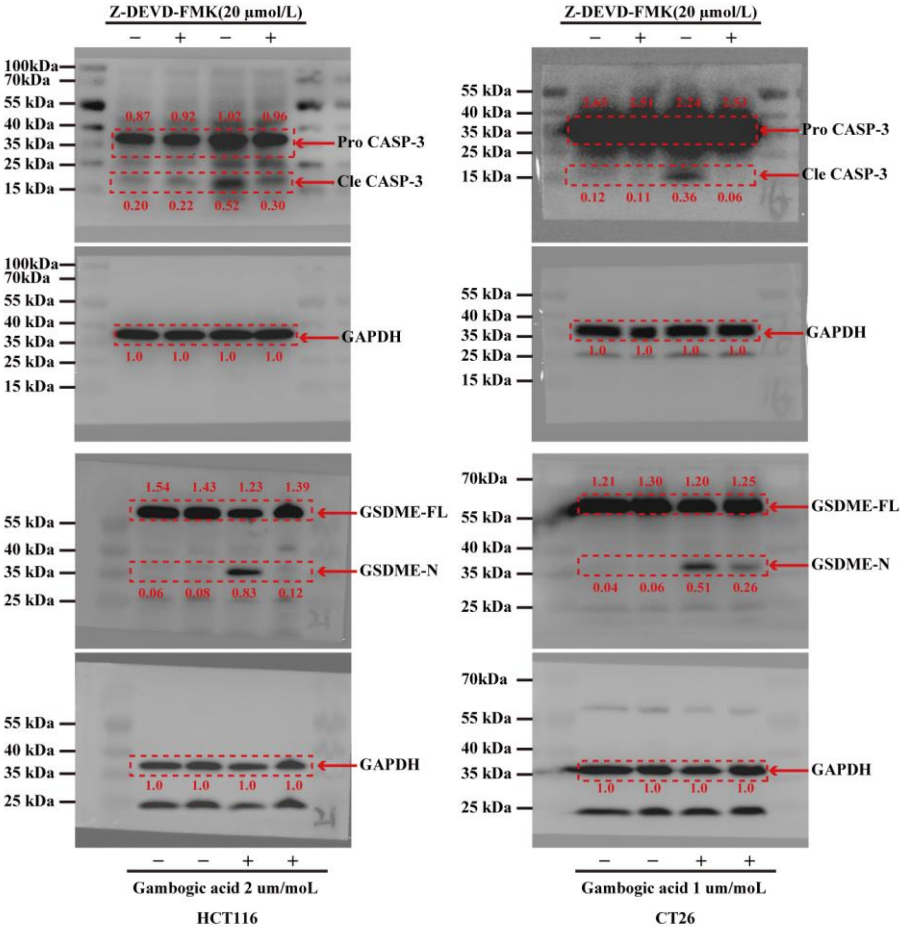

Figure S5. Original western blots of Figure 6B.

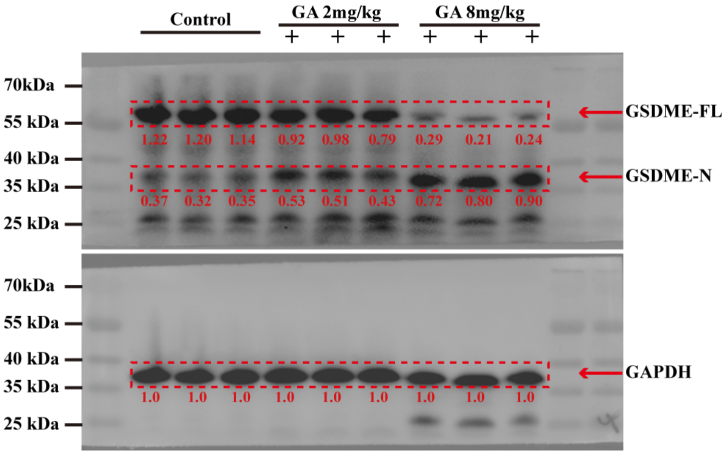

Figure S6. Original western blots of Figure 7E.
